# UV-Absorption—The Primary Process in Photocatalysis and Some Practical Consequences

**DOI:** 10.3390/molecules191118192

**Published:** 2014-11-06

**Authors:** Terry A. Egerton

**Affiliations:** School of Chemical Engineering and Advanced Materials, University of Newcastle, Newcastle NE1 7RU, UK; E-Mail: Terry.Egerton@ncl.ac.uk or tpj.egerton@virgin.net; Tel.: +44-(0)1642-645-732

**Keywords:** UV, photocatalysis, photochemistry, semiconductor, TiO_2_, rutile, anatase, absorption, scattering, particle-size

## Abstract

TiO_2_ photochemistry studies generally address reactions of photogenerated charge-carriers at the oxide surface or the recombination reactions which control the proportion of charge carriers that reach the surface. By contrast, this review focuses on UV absorption, the first photochemical step in semiconductor photocatalysis. The influence of particle size on absorption and scattering of light by small TiO_2_ particles is summarized and the importance of considering, the particle size in the application, not the BET or X-ray line broadening size, is emphasized. Three different consequences of UV absorption are then considered. First, two commercially important systems, pigmented polymer films and paints, are used to show that TiO_2_ can protect from direct photochemical degradation. Then the effect of UV absorption on the measured photocatalytic degradation of aqueous solutions of organics is considered for two separate cases. Firstly, the consequences of UV absorption by TiO_2_ on the generation of hydroxyl radicals from H_2_O_2_ are considered in the context of the claimed synergy between H_2_O_2_ and TiO_2_. Secondly, the effect of altered UV absorption, caused by changed effective particle size of the catalyst, is demonstrated for photocatalysis of propan-2-ol oxidation and salicylic acid degradation.

## 1. Introduction

Semiconductor photocatalysis by TiO_2_ has been widely studied for 50 years. Potential uses include destruction of bacteria [1], the oxidation of pollutants [2], e.g., dye residues [3], and removal of organic films from glass and polymer substrates [4]. However, early work emphasized undesirable aspects, e.g., photocatalytic degradation of TiO_2_ pigmented paint films [5] or textile fibres [6]. Commercial research to minimize TiO_2_ photocatalysis continues, but little is published in the open literature. Both objectives have driven research into the photocatalytic mechanism [7,8]. It is generally agreed that UV absorption excites an electron from the valence band to the conduction band of the semiconductor. The resulting excited electrons, in the otherwise empty conduction band, and the “positive holes” in the valence band allow charge transfer to the TiO_2_ surface which facilitates oxidation of surrounding molecules. Sometimes direct charge-transfer causes the oxidation [9]. Alternatively, hydroxyl radicals, formed by the “positive holes” in the valence band accepting electrons from hydroxyl ions, are the catalytically active intermediates [8,10].

The *Grotthus-Draper Law*, formulated in 1817 and rediscovered in 1841, has been stated as “*Only radiations which are absorbed by the reacting system are effective in producing chemical change.*” [11]. However, discussion of light absorption in semiconductor photocatalysis is generally restricted to extending absorption to longer wavelengths (to harvest a greater proportion of solar radiation) [12] and the possibility of photo-sensitizing reactions by exploiting electron transfer to the semiconductor from excited states of adsorbed dyes [13]. UV-photocatalytic papers mainly address topics such as the activity of new TiO_2_ preparations [14,15], the intermediates formed in specific reactions [16], the extent to which transition metals modify charge-carrier recombination [17,18], and whether specialized procedures such as the application of an electric field increase photocatalytic activity [19]. Often, potential changes in UV-absorption of the particle dispersion resulting from, e.g., the preparation of novel TiO_2_’s, or from supporting catalysts on inert supports or from inadvertently changing particle aggregation during surface modification or doping, are overlooked. Therefore this survey focuses on ways in which unconsidered aspects of UV-absorption may modify either undesirable or desirable photocatalysis.

## 2. A Summary of Small Particle Optics (Where Diameter, d, Is Comparable with the Radiation Wavelength, λ)

### 2.1. Background Theory

**Figure 1:**
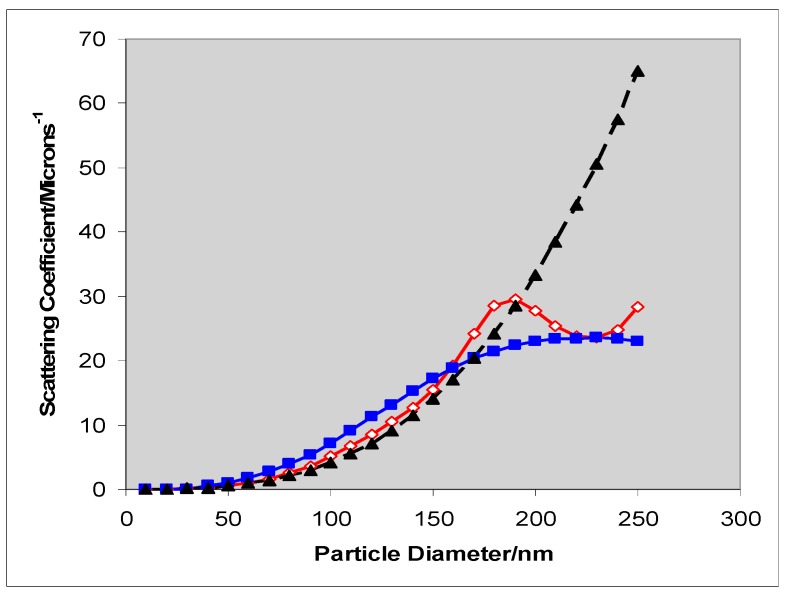
Rayleigh (▲, dashed line) and Mie scattering (**◊** and ■, full lines) of 555 nm radiation by isolated rutile spheres as a function of particle diameter. The curves plotted through ▲ and **◊**, points assume that all particles are of identical. The curve plotted through the ■ points assumes a log-normal distribution of particle size.

The optical properties of particulate dispersions, unlike those of molecular solutions, depend on absorption and scattering. For particulate suspensions the well known extinction equation may be written as:

log_10_(I_o_/I_t_) = q.c.l = (q_abs_ + q_sca_ ) (1)

where c is the concentration of particles in suspension and l is the path length and q, the extinction coefficient, is the sum of the coefficients of scattering, q_sca_, and absorption, q_abs_. Scattering of radiation, of wavelength λ, by small particles, of diameter d (d/λ << 0.1), was analyzed by Rayleigh [20]. Later Mie’s treatment of scattering by larger spherical particles took into account interference between light scattered from different points on the particle surface [21]. The difference between Rayleigh scattering and Mie scattering of 555 nm radiation by isolated, spherical, rutile particles is shown in Figure 1 (both theories describe optically dilute systems, *i.e.*, neither treats interference between light scattered by *different* particles.)

**Figure 2:**
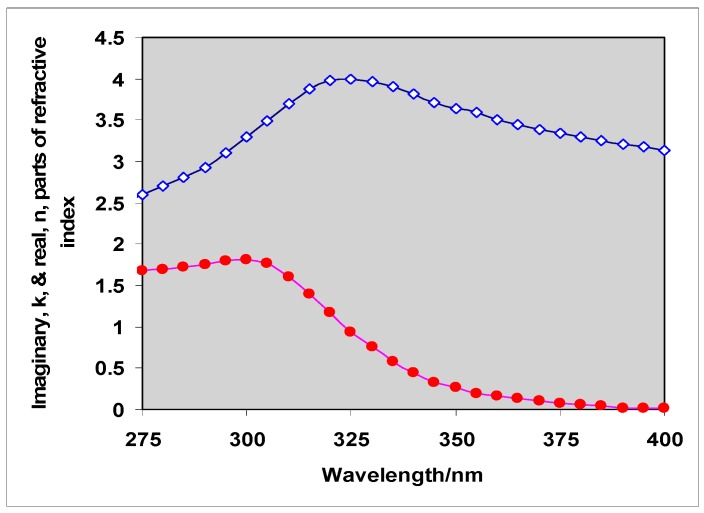
Variation of the real, ◊, and imaginary, ●, components of the refractive index of rutile between 275 and 400 nm based on results of Vos and Krusmeyer [22]. Above 400 nm the imaginary component, which controls light absorption, is negligible even though the real component is not. Pure rutile crystals are transparent in the visible region of the spectrum but can scatter visible radiation.

**Figure 3:**
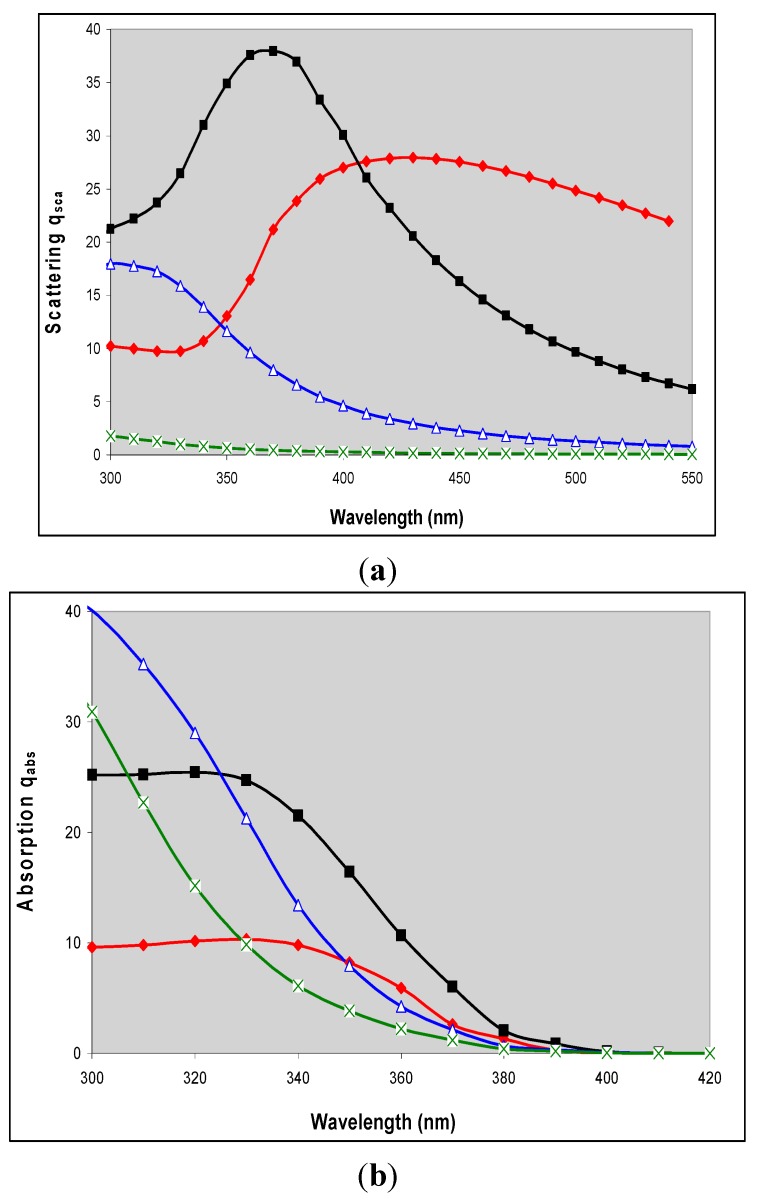
Mie theory calculations of (**a**) q_sca_, scattering and (**b**) q_abs_, absorption per unit volume for 20 nm, ×; 50 nm, Δ; 100 nm ■; and 220 nm ♦ rutile particles dispersed in an organic medium, plotted as a function of mean size for a log normal particle distribution with σ = 1.33. Reproduced with permission from Egerton & Tooley, International Journal of Cosmetic Science **2012**, *34*, 117–122 [23] published by Society of Cosmetic Sciences; Société Française de Cosmétologie and Blackwell Publishing.

Mie showed that light scattering depends on both the ratio d/λ, and on the refractive index of the medium, m_m_, and the particle, m_p_. Refractive index is a complex quantity with real and imaginary components and, as shown for rutile spheres, in Figure 2 both of these vary with wavelength. Light *scattering* is controlled by the real component; light *absorption* by particles is controlled by both real and imaginary components and (unlike absorption by molecules) also by particle size. It is often convenient to express both scattering and absorption on a unit-volume (or unit-mass) basis and typical results are shown in Figure 3a,b [23].

Calculated scattering and absorption coefficients of a particulate suspension are shown in Figure 3a,b. The extinction coefficient may be calculated by simply summing the scattering and absorption coefficients. Inspection of these graphs shows that in an organic medium the percentage contributions of absorption to the extinction coefficient of for mean sizes of 20, 50, 100 and 220 nm particles are 94% (93%), 66% (63%), 53% (52%) and 49% (46%) at 310 nm and are 81% (50%), 30% (17%), 22% (14%) and 26% (30%) at 360 nm. The results in brackets are the results of earlier calculations published by Robb, Simpson and Tunstall [24] (the differences between the two sets are probably a consequence of small differences in the input refractive indices and the size-distributions in the two sets of calculations). Except for 20 nm particles at a wavelength of 360 nm the results of the two sets of calculations are in reasonable agreement. Both sets show that the contribution of absorption is greater at 310 than at 360 nm for all sizes in the range 20 to 220 nm, but even at 310 nm, the absorption contribution becomes progressively less important as the mean particle size is increased. For 220 nm particles, the size of a typical TiO_2_ pigment, scattering and absorption make approximately equal contributions to attenuation of 310 nm radiation but at 360 nm the most important contribution to attenuation is scattering. Because both the absorption and scattering are particle-size dependent absorption and scattering coefficients from, e.g., measurements on single crystals [25], on CVD films [26], or from study of different TiO_2_ particles [27] cannot be used to calculate the radiation flux in a photocatalytic study.

### 2.2. Comparison of Theory and Experiment

**Figure 4:**
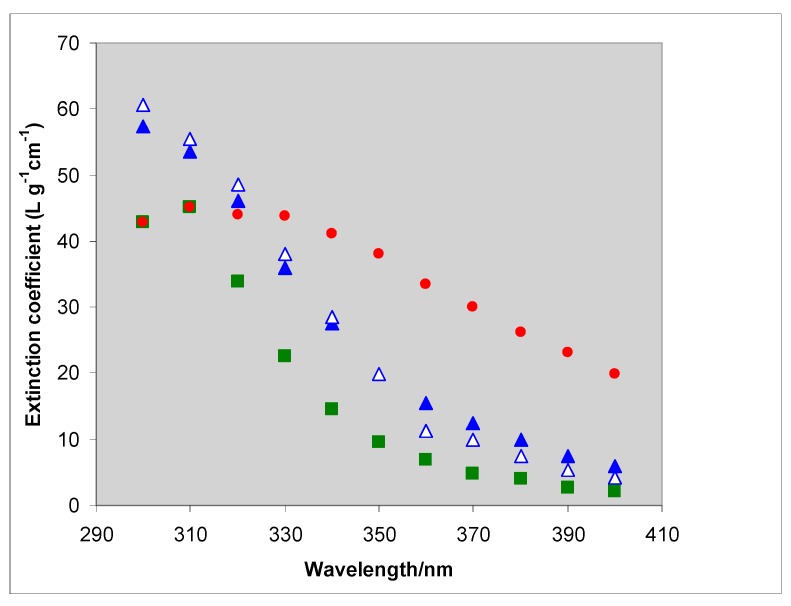
Experimental extinction coefficients for 35 ■, 50 ▲ and 145 nm ● rutile particles and the calculated coefficients for 50 nm ∆, derived from Figure 3a,b.

The variation in absorption and scattering coefficients with the ratio of d/λ is paralleled by changes in the angular distribution of the scattered light [28]. Consequently the experimental estimation of the amount of radiation absorbed by a particulate suspension is not straightforward. In a typical “optical-density” type transmission measurement, as used in most photocatalytic studies, the measured attenuation is the sum of the absorption and the variable fraction of light not incident on the detector because it is scattered out of the beam. A proper measure of the radiation absorbed by the TiO_2_ requires the amount total amount of scattered light to be measured—by the use of an integrating sphere [23,29]. Figure 4 compares calculated (from the results in Figure 3a,b) and experimental coefficients for 50 nm rutile and shows how the experimental result changes as size (measured by x-ray sedimentation) varies from 35 to 145 nm [23]. A comparison with the absorption properties of sunscreen formulations has also been made [23]. Satuf *et al.* have used diffuse reflectance and transmittance spectrophotometric measurements of suspensions to derive curves for Aldrich TiO_2_, Degussa (now Evonik) P25 and Hombikat UV 100. At 310 and 360 nm the derived values of absorption coefficient are ca 3.1 and 4.6 × 10^4^ cm^2^·g^−1^ respectively for P25 and 0.75 and 0.5 for Hombikat UV 100 [29]. When the scattering and absorption coefficients are known, they may be used to identify optimum catalyst loading for reactors of specified geometry and radiation input [30]. Li Puma *et al.* have developed a 6-flux model, to estimate the flux throughout a photocatalytic reactor and have used their treatment to model the effect of catalyst loading on the overall volumetric rate of photon absorption, and hence on the effective quantum yield [31,32].

**Figure 5:**
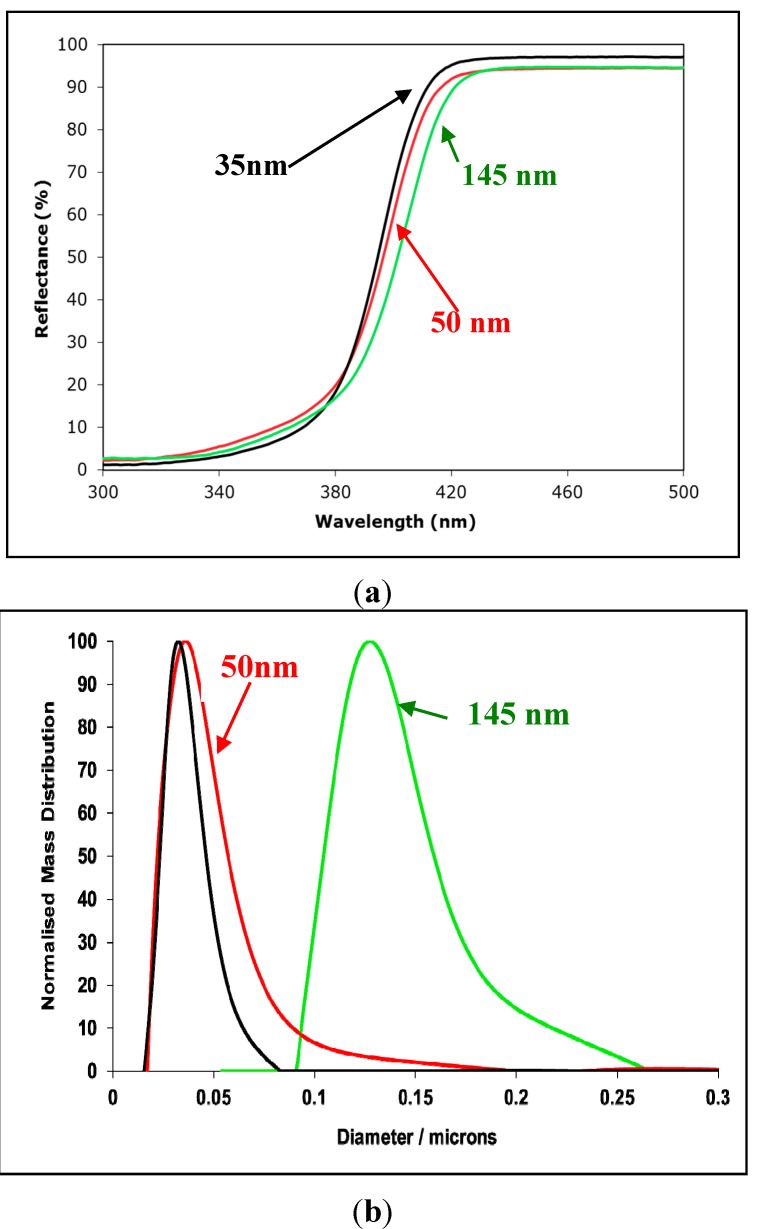
(**a**) The reflectance spectra (measured on a Jasco 670 spectrometer fitted with an integrating sphere) of pressed discs made from the three different rutile samples of mean size 35 ─; 50 ─ and 145 ─ whose suspension spectra are shown in Figure 4 and whose size distributions are shown in Figure 5b; (**b**) The particle-size distributions measured by X-ray size sedimentation (Brookhaven X-ray disc sedimentometer) of three rutile samples (reprinted with permission from Egerton & Tooley, International Journal of Cosmetic Science **2012**, *34*, 117–122 [23] published by Society of Cosmetic Sciences; Société Française de Cosmétologie and Blackwell Publishing).

Although conventional reflectance spectra on pressed discs of powder may usefully demonstrate the position of the absorption edge (or band-gap) [12], and confirm, for example, that anatase absorbs at shorter wavelengths (has a larger band-gap) than rutile, they do not effectively show differences in absorption at wavelengths shorter than the band gap by suspensions of particles. This is illustrated in Figure 5a which shows the very similar diffuse reflectance spectra of compacts of three rutile samples with mean sizes of 35, 50 and 145 nm, and the size distributions shown in Figure 5b, whose extinction coefficients in suspension are shown in Figure 4. Even though there are large differences between the transmission spectra of suspensions of the three samples, the differences between the reflectance spectra are negligible. Little information about the optics of particle suspensions is contained in the reflectance spectra of discs made from powders from which the suspensions were prepared.

Although conventional reflectance spectra on pressed discs of powder may usefully demonstrate the position of the absorption edge (or band-gap) [12], and confirm, for example, that anatase absorbs at shorter wavelengths (has a larger band-gap) than rutile, they do not effectively show differences in absorption at wavelengths shorter than the band gap by suspensions of particles. This is illustrated in Figure 5a which shows the very similar diffuse reflectance spectra of compacts of three rutile samples with mean sizes of 35, 50 and 145 nm, and the size distributions shown in Figure 5b, whose extinction coefficients in suspension are shown in Figure 4. Even though there are large differences between the transmission spectra of suspensions of the three samples, the differences between the reflectance spectra are negligible. Little information about the optics of particle suspensions is contained in the reflectance spectra of discs made from powders from which the suspensions were prepared.

At least two factors contribute to the difference between the reflectance and transmission spectra. Firstly, the reflectance spectra depend on the ratio, K/S, of the absorption coefficients (K) and the scattering coefficient (S) of the powder. Since both of these depend on the ratio d/λ the effect of changes in particles-size, d, are minimized (by contrast, the transmission spectrum of Figure 4 depends on the sum of K and S). Secondly, because of interference effects, the scattering of particles is strongly influence by particle-particle distance, which is clearly quite different in dilute suspensions and pressed powder compacts [33].

### 2.3. The Major Problem Associated with the Calculation of Attenuation by Semi-Conductor Dispersions

**Figure 6:**
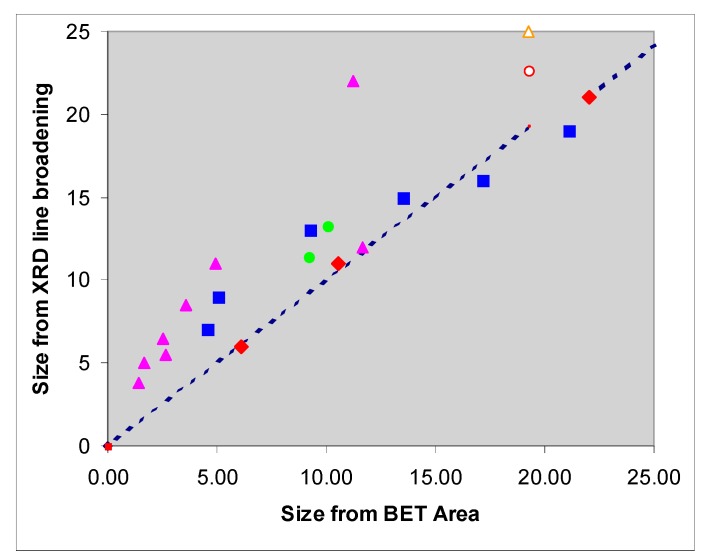
A comparison of published XRD sizes with the BET-derived sizes of (mainly) anatase samples identified from the publications listed in the caption to figure 15 of Egerton, T.A.; Tooley, I.R., *Intl. J. Cosmetic Sci*. **2014**, *36*, 195–206 [35]. The dashed line corresponds to S = 6D/ρ. where S is the surface area, D the particle diameter and ρ the particle density.

The previous sections have shown that scattering and absorption by TiO_2_ particles depends on their size in suspension. The size of the fundamental, or primary, particles may be measured by X-ray line broadening (if the particles are crystalline) or inferred from BET surface area measurements, using area gm^−1^, A = 6 d/ρ), and are usually in reasonable agreement (Figure 6) but it is rare for the TiO_2_ to exist as isolated primary particles in suspension.

**Figure 7:**
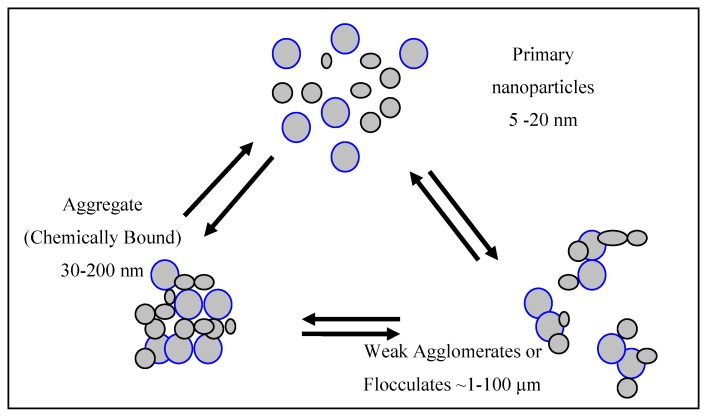
Schematic representation of the aggregation and agglomeration of titanium dioxide nanoparticles. Primary particles may flocculate to form weakly bound agglomeratesor sinter to form much stronger aggregates. Agglomerates may sinter to form more strongly bound aggregates. Agglomerates break down more easily than aggregates.

Instead, as represented schematically by Figure 7 and, for the case of P25, by the transmission micrographs of Figure 6 of reference [34], the particles in typical photocatalytic suspensions are, if great care has been taken, aggregates, but more usually, weakly-bound agglomerates. (By agglomerates is meant a secondary particle composed of flocculated or coagulated particles which can be broken down by changes in such factors as suspension pH and mechanical forces. By Aggregate is meant a more strongly bound secondary particle—perhaps formed by sintering during the particle preparation process—and much less susceptible to mechanical disruption.)

Since the gap between the primary particles is normally small in comparison with the radiation wavelength, the optical behaviour is controlled by the size of the aggregates or agglomerates in suspension as explicitly recognized by both Martin, Baltanas and Cassano [34] and by Egerton and Tooley [36]. If the pH is near the isoelectric point there is negligible particle-particle electrostatic repulsion but significant particle attraction because of TiO_2_’s high Hamaker constant. On the basis of electron microscopy Martin *et al.* concluded that “*Any degree of stirring (be it mechanical agitation, pump recycling or gentle gas bubbling) produces an important change in the optical properties*” [34]. However, because the primary particle size *is* relevant to calculations of adsorption and surface-coverage, and perhaps because the primary particle size is easier to measure, particle characterization continues to be mainly by surface area and XRD derived sizes. Therefore, it is appropriate to summarize some of the results which show that there is normally significant particle agglomeration in suspension, even at pH’s at which there is significant surface charge.

Ridley and coworkers studied 0.15 g·dm^−3^ aqueous dispersions of a crystalline anatase (Ishihara ST-01) at a pH of 2.7, well below the p.z.c. of 6.85 [37]. The TEM size was 4.6 nm, the BET equivalent sphere diameter was 5.0 nm, and the XRD crystallite size was 7 nm. However, laser diffraction methods showed the dispersed particles to have a relatively small mode centred near 100 nm and to consist predominantly of microscale aggregates with a median diameter of 2329 nm (99.6% of the distribution volume was characterized by diameters greater than 400 nm). At this pH, ultrasonication did not significantly alter the measured size distribution. French and co-workers used dynamic light scattering (DLS) at a sample concentration of ~40 mg·dm^−3^ to show that 4–5 nm sol-gel derived anatase particles form stable agglomerates with an average diameter of 50–60 nm at pH ~ 4.5 in 0.0045 M NaCl [38]. When the ionic strength was increased to 0.0165 M micron-sized agglomerates formed within 15 min. At all other pH values tested (5.8–8.2), micron-sized agglomerates formed in less than 5 min even at low ionic strength (0.0084–0.0099 M NaCl). DLS measurements were used by Lee *et al.* to show that in suspension particles with a TEM size below 100 nm, increased in size from ~100 to 500 nm as their concentration increased from 5 to 80 mg·dm^−3^ [39]. Egerton and Tooley compared XRD line-broadening and, BET equivalent sphere diameters with sizes measured by X-ray sedimentation and DLS of three surface-treated rutile samples [35]. In each case the DLS size was and 5–10 times larger than the XRD or BET sizes. The sedimentation size and DLS sizes were comparable. A comparison of XRD, BET, sedimentation and Laser measurements is shown in Table 1.

The size-measurement results are, with hindsight, supported by two widely used experimental procedures—filtration of catalysts from reaction mixtures and the practice of stirring suspensions of photocatalysts to prevent sedimentation. If the particles were dispersed, it would be impossible to remove 20 nm particles of P25 catalyst particles from reaction mixtures by 0.1 μm Millipore filters, or by guard columns, as is commonly done prior to chromatographic analysis [3,36]. Also, since Stokes’ equation shows that primary particles would not sediment significantly over the time of a typical catalytic experiment, there would be no need of the common experimental practice of stirring suspensions of photocatalysts to prevent sedimentation [2,40]. Despite this problem, it is still both useful and important, to consider the effect of absorption when interpreting the results of photocatalytic experiments by dispersions of TiO_2_. The rest of this paper will give some examples of how this may be the case.

**Table 1:** A comparison of particle-sizes of nano-particulate TiO_2_ measured by X-ray line broadening or by nitrogen adsorption (BET) on the dry powders and by X-ray sedimentation and laser diffraction or laser scattering on aqueous dispersions of TiO_2_.

Base Crystal & Surface-Treatment	XRDLine Broadening	Equivalent Sphere Diameter from BET Area	SedimentationBrookhaven	Dynamic Light Scattering (DLS) or Laser Diffraction
1. Ishihara ST-01 Anatase Untreated [37]	7	5	-	2329 Beckman Coulter LS-230 Laser Diffraction
2. Rutile-A silica-alumina [35]	15	12	91 ± 10	133 (DLS)
3: Rutile-A Stearate [35]	15	23	53 ± 10	124 (DLS)
4: Rutile-B Stearate [35]	9	48	160 ± 19	201 (DLS)

## 3. The Need to Consider UV Absorption when Interpreting Photocatalytic Results

### 3.1. Protection against Photodegradation of Organic Materials by Dispersed TiO_2_

#### 3.1.1. Photodegradation of Polyethylene Films

**Figure 8:**
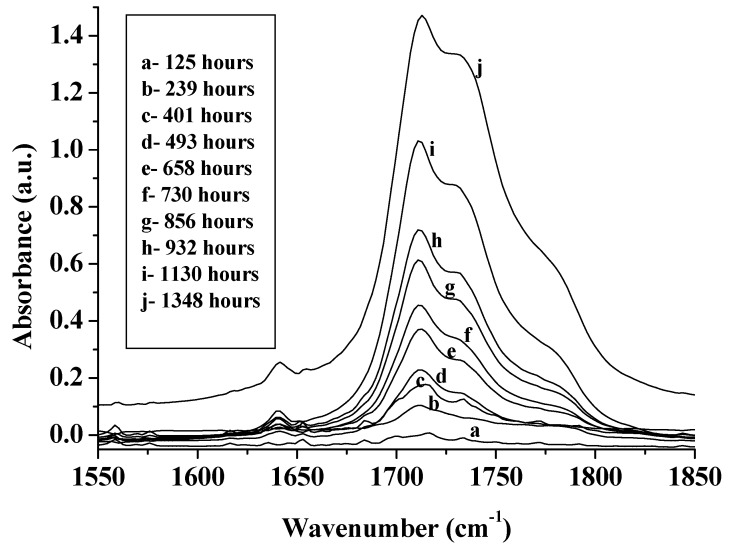
The development of the infrared absorption characteristic of carbonyl oxidation products in an unpigmented polyethylene film as the UV exposure in QUV accelerated weathering equipment, fitted with UVA-340 tubes and operated at 40 °C, increases from 125 to 1348 h. (reprinted from Polymer Degradation and Stability. **2007**, *92*, 2163–2172 [41] with permission).

Academic studies of photocatalysis usually select systems in which oxidative degradation is negligible in the absence of TiO_2_. However, many commercial systems—e.g., most polymers and paints are degraded by UV even when TiO_2_ is not present and Figure 8 shows the increasing infrared signature of the carbonyl oxidation products of an unpigmented polyethylene (PE) film exposed to UV for increasing times [41]. Ultimately the film is totally oxidized to carbon dioxide and water.

**Figure 9:**
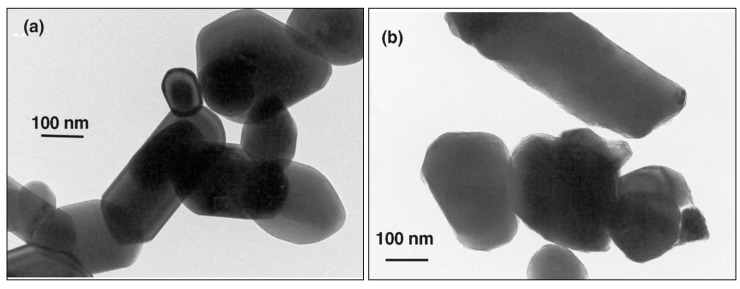
Transmission electron micrographs showing (**a**) the uncoated TiO_2_ crystals and (**b**) surface treated (coated) TiO_2_ rutile pigment. The ZrO2/Al2O3 coating, with a thickness of 3–10 nm, shows as a less dense outline to the images (reprinted from J. Mater. Sci. **2002**, *37*, 4901–4909 [47] with permission).

If TiO_2_ is present, it has normally been added to opacify the product—e.g., to pigment a polythene film. Commercial pigments, often rutile because its higher refractive index, enhance opacity, and are frequently surface-treated, e.g., with an amorphous layer of hydrous silica and/or alumina and or/zirconia (see Figure 9) to minimize photocatalysis, and lengthen the lifetime of the product [5,42,43,44,45,46].

**Figure 10:**
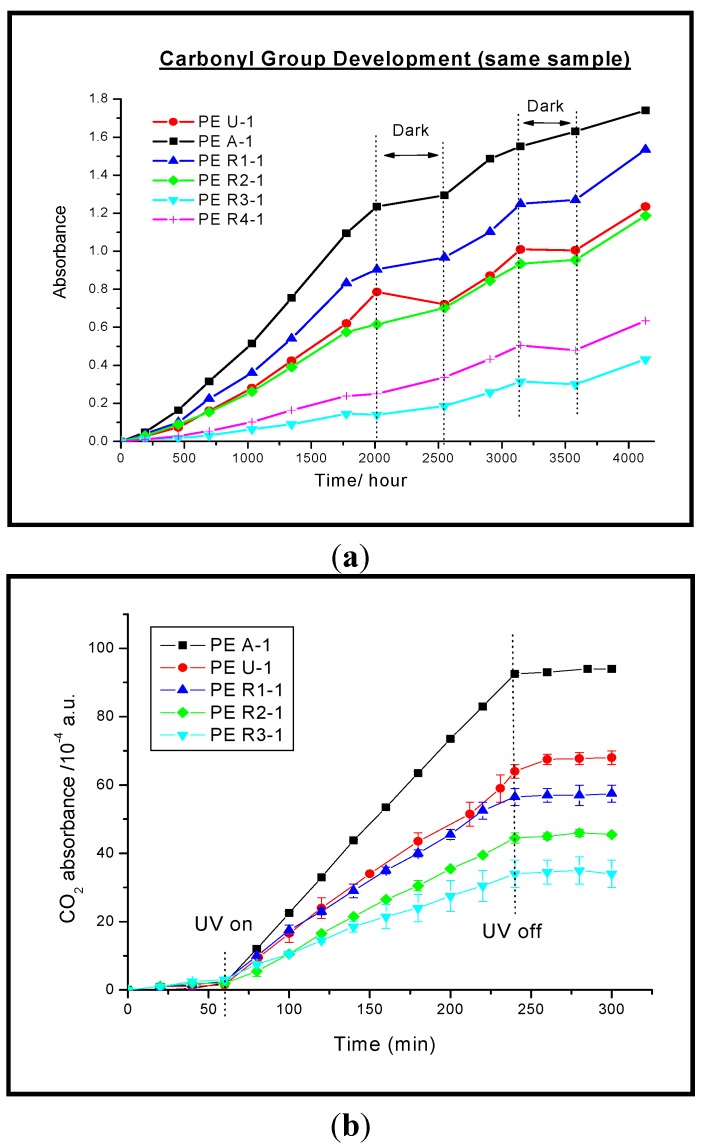
(**a**) The development of carbonyl absorption in unpigmented and pigmented polythene films (PE A-1, ■; PE R1-1, ▲, PE U-1, ●; PE R2-1, ♦; and PE R3-1, ▼ and PE R4-1, ┼. as a function of exposure in QUV accelerated weathering equipment. After recording each IR spectrum the disc was returned to the exposure unit for further UV exposure); (**b**) CO_2_ evolution from the photo-oxidation of the same films as used for the carbonyl development measurements in Figure 10a but exposed to irradiation from a xenon lamp [46].

In polymers pigmented with a surface treated rutile, the absorption of UV by added TiO_2_ may reduce the photochemical oxidation and extend the life of the polymer and this is shown in Figure 10 where the development of the carbonyl absorption in 100 μm thick films of low density polyethylene in unpigmented (PE-U1) and films prepared with different TiO_2_ pigments at a loading of 5 p.h.r. (parts per hundred resin by weight). For the anatase (PE-A1) and lightly coated rutile (PE-R1-1) films incorporation of pigment has increased the photodegradation—*i.e.*, photocatalysis by the TiO_2_ is more important than any UV absorption by the pigment. For the remaining pigments (R3-1 and R4-1), in which photocatalysis has been suppressed by a heavier surface coating UV absorption, the absorption by UV dominates so that the total degradation is less than that of the unpigmented film. This reduction occurs even though the total path length is only 100 μm and at wavelengths greater than 250 nm the absorbance is less than 0.2 for the unpigmented film. Figure 10b shows that the same pattern is observed if total oxidation of the polymer to carbon dioxide is measured instead of the formation of the intermediate carbonyl groups [41]. A similar pattern is also observed in PVC for which Worsley *et al.* have noted that the activity of P25 is two orders of magnitude higher than that of a Al/Si/Zr coated rutile pigment [45].

#### 3.1.2. Photodegradation of Alkyd Paint Films

**Figure 11:**
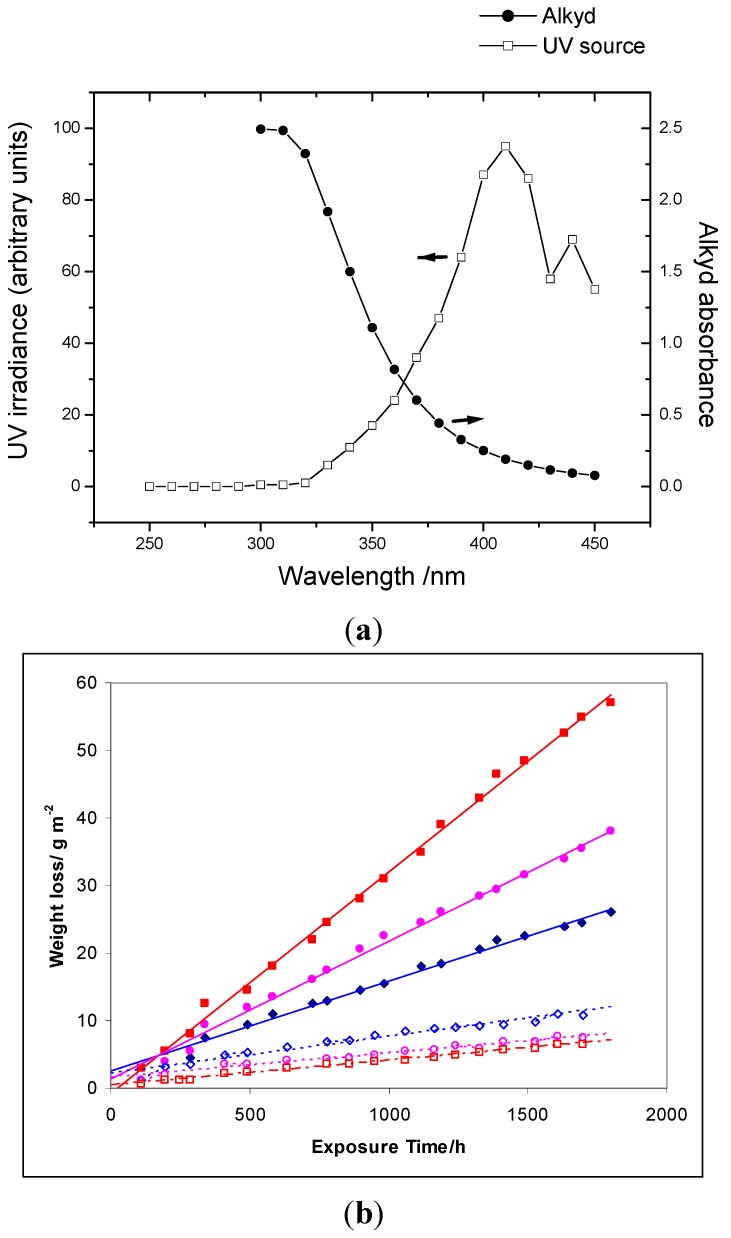
(**a**) UV absorption of a 90 μm unpigmented alkyd film compared with the spectral distribution of the carbon arc lamps used in an “accelerated weathering test”. LH scale, arc lamp distribution; RH scale, Film Absorbance (reprinted from *J. Mater. Sci.* **2002**, *37*, 4901–4909 [47] with permission); (**b**) The degradation (weight loss as a function of time exposed to radiation from carbon arcs) of paint films of long-oil soya alkyd opacified with either uncoated rutile (solid points) or coated rutile (empty points) by 40 (■,□), 25 (●○) and 5 (♦ ◊) volume % pigment.

Alkyd films, of the type used in oil-based paints, absorb more strongly between 300 and 400 nm (Figure 11a) than PE films, and the pigment volume fraction (p.v.c.) of TiO_2_ in an alkyd paint may be ten times that in a polymer film. The oxidative degradation of these paints to CO_2_ may be measured by monitoring their loss in weight when exposed to UV and Figure 11b shows results for a series of such paints exposed to UV in carbon-arc Marr weathering equipment [47]. For the uncoated rutile the weight-loss increases as the p.v.c. increases from 5% to 40%. The much lower weight loss from paints made with coated pigment confirms that the surface coating reduces the pigment photoactivity and therefore has increased the relative importance of protective UV absorption. The decrease in weight loss as the p.v.c. of the coated pigments increases from 5% to 40% demonstrates that the protective role of TiO_2_ can also occur when the TiO_2_ loading is much higher than that in polymer films.

#### 3.1.3. Disinfection by UV-C

Dillert, Siemon and Bahnemann compared the disinfection of: (a) pre-treated municipal wastewater and (b) a model pollutant, *E. coli.* [48] by two different lamps, A and B. In the wavelength ranges < 280 nm, 280–315 nm, and 315–400 nm the output of lamp A (Solidex glass envelope) was 5, 150 and 220 mEinstein·h^−1^ whilst that of B (Jena glass envelope) was 0.2, 18 and 390 mEinstein·h^−1^, respectively. *i.e.*, the short wavelength output of A was much greater than that of B. A summary of their results (Table 2) shows that although, without TiO_2_, both lamps disinfected, A, because of its higher UV-B and UV-C output, was more effective than B in both systems. However, addition of 5 g·dm^−3^ P25 TiO_2_ reduced the effectiveness of lamp A (disinfection times increased to more than 360 min. for waste-water and to more than 270 min. for *E. coli*) but greatly increased the effectiveness of lamp B for disinfection of *E. coli* and this was attributed to photocatalysis by the TiO_2_. By contrast with this increase, the effectiveness of lamp B for wastewater treatment was decreased. Dillert *et al.* attributed the significant reduction of the effectiveness of lamp A to the “shadowing effect” of the TiO_2_ particles. Their conclusion that photon absorption by TiO_2_ can reduce the number of photons available for direct photochemical not only demonstrates the importance of UV absorption in the photocatalytic treatment of practical systems, but also implies that this absorption may be more important with respect to some wavelengths (in this case short wavelengths) than for others.

**Table 2:** A comparison, derived from the results of Dillert *et al.* of the disinfection achieved by two different UV lamps, A and B [48]. The results show the potential importance of UV absorption by TiO_2_ in reducing the disinfection rates in systems for which direct UV treatment is effective.

Conditions	Pre-Treated Wastewater		*E. coli*	
	Initial Number of Colony Forming Units (c.f.u.)	C.f.u after 60 min Treatment	Initial Number of Colony Forming Units (c.f.u.)	C.f.u after 60 min Treatment
Lamp A	~6 × 10^4^	Not detectable	3 × 10^6^	Not detectable
Lamp A+TiO_2_	3 × 10^5^	1.5 × 10^4^	6 × 10^6^	1 × 10^5^
Lamp B	1 × 10^4^	2 × 10^2^	~8 × 10^6^	~6.5 × 10^5^
Lamp B+TiO_2_	3 × 10^4^	6 × 10^3^	~8 × 10^6^	1.5 × 10^1^

### 3.2. Reduced Photocatalytic Activity of Other Solution Species because of UV Absorption by Nano-Particulate TiO_2_

**Figure 12:**
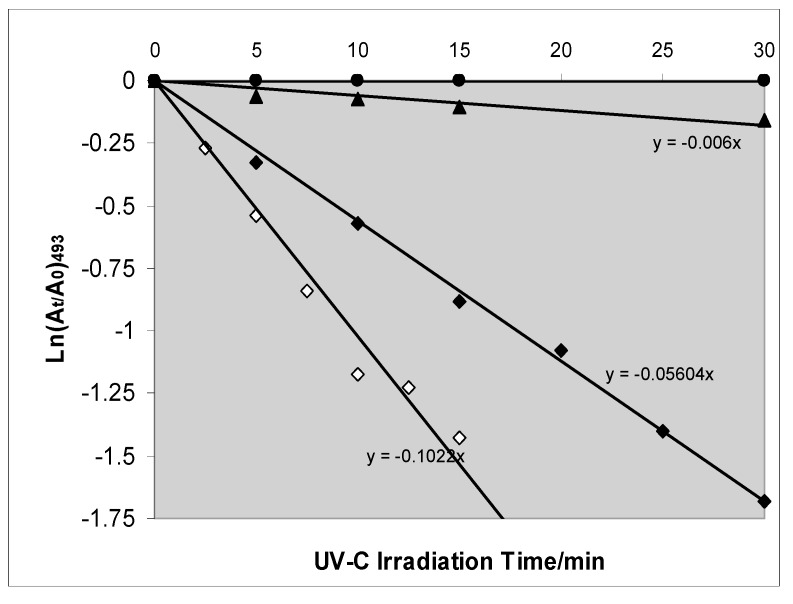
UV-C decolouration of 0.05 mM RO16 in the presence of TiO_2_ (● UV-C only: ▲ 2 g·dm^−3^ TiO_2_: ♦ 2 g·dm^−3^ TiO_2_, 20 mM H_2_O_2_: ◊ No TiO_2_, 20 mM H_2_O_2_). Reprinted from Egerton, T.A.; Purnama, H. *Dyes Pigments* **2014**, *101*, 280–285 [53] with permission.

UV-C photocatalysed dye-decolouration by hydrogen peroxide/anatase mixtures has been widely reported to be faster than photocatalytic decolouration by anatase alone. For Tropaeoline and Reactive Red the reaction rate is doubled [49,50]; for Safira HEXL anionic azo dye the rate is enhanced ×4.5, at pH 7, and ×9, at pH 5 [51]. For Acid Red 14 a 20 fold enhancement has been reported [52]. However results such as those shown in Figure 12, for the UV-C decolouration by anatase PC500 of the azo-dye Reactive Orange 16, suggest that the true picture is less simple [53]. Experiments with both 2 mM and 20 mM H_2_O_2_ showed that although decolouration by UV-C+TiO_2_+H_2_O_2_ is faster than decolouration by UV-C+TiO_2_, it is slower than decolouration by UV-C+H_2_O_2_ in the absence of anatase. If the reaction is carried out in water TiO_2_ the rate is decreased to 45% of that H_2_O_2 _ alone. In 0.1 M NaCl the reduction is by 43% and In 0.1 Na_2_SO_4_ the reduction is 64%. UV-C photolyses H_2_O_2_ to hydroxyl radicals with a quantum efficiency that can approach unity [54] but the quantum efficiencies for the TiO_2_-photocatalysed processes are << 5%. Therefore, the reduction in decolouration rate when TiO_2_ is added to H_2_O_2_ shows that UV-C photon absorption by the TiO_2_ reduces the number of photons which would otherwise be available to efficiently photolyse H_2_O_2_.

### 3.3. Changes in UV Absorption Caused by Agglomeration or Flocculation Alter the Measured Photocatalytic Activity of Nano-Particles

#### 3.3.1. Predicted Change in Photocatalytic Activity Resulting from an Increase in UV Absorption

As stressed in Section 2.3, because of flocculation/agglomeration the effective size of TiO_2_ particles in suspension is larger than that of their constituent primary particles. As illustrated by the large flocculate sizes measured by DLS and other optical techniques (Table 1), it is the size of the secondary particles that control the optics (because UV photons have a much larger wavelength, ~300 nm, than the spaces between the primary particles (10–20 nm). However, because charge transfer between primary particles is limited, the charge-carrier recombination, unlike the optical behaviour, is limited mainly by the size of primary particles. Consequently, changes in the degree of particle agglomeration, such as brought about by milling, should change the measured photocatalytic activity.

**Figure 13:**
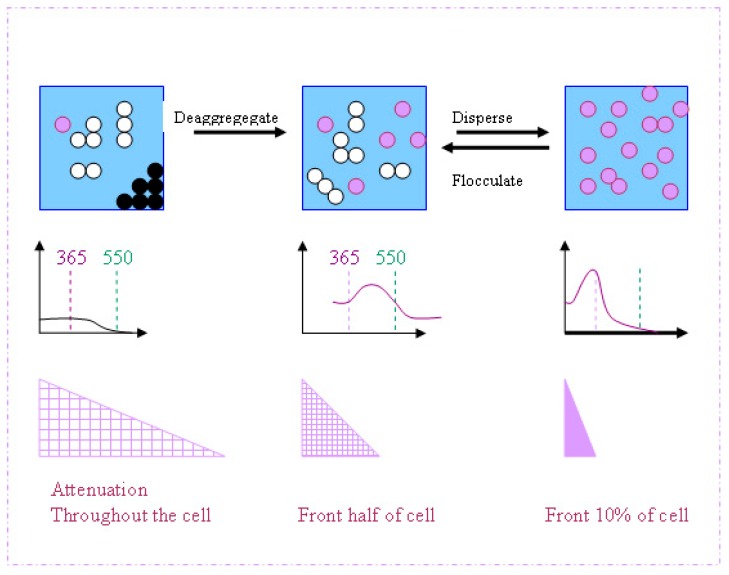
A schematic depiction of the effects of increased UV absorption associated with improved dispersion of TiO_2_ particles. The second and third rows represent changes in the transmission spectrum and the attenuation of the incident UV beam as the particle dispersion is altered in the way depicted in the top row.

The absorption curves of Figure 3b demonstrate that UV-A absorption increases significantly as rutile particle size is decreased from 200 to 100 nm. Assuming that the reactor geometry is such that all the UV is absorbed, the effect of increased absorption is that the same amount of UV is absorbed in a shorter path-length—by fewer primary particles, as illustrated by Figure 13.

The effect of this difference in absorption may now be considered. In a suspension each primary particles acts as micro-reactor and the flux of incident photons is usually sufficiently large for recombination kinetics to dominate, *i.e.*, the rate is proportional to the square root of incident intensity [8,29,55,56,57].

Then, if all of the P incident photons are absorbed by n of the more weakly absorbing 200 nm particles, generating P/n electron-hole pairs in each, the observed rate, R_200_, is proportional to the product of the number of micro-reactors and the rate in each of them:

R_200_ = k × n × (P/n)^0.5^ = k × P^0.5^n^0.5^ (2)

If the 100 nm particles absorb twice as strongly as the 200 nm particles all of the photons will be absorbed in only n/2 particles and the number of electron-hole pairs in each of these will be P/(n/2) = 2P/n. This leads to an observed rate, R_100_, given by:

R_100_ = k × n/2 × (2P/n)^0.5^ = k × P^0.5^n^0.5^2^−0.5^ (3)

The effect of the *increased* absorption has been to *decrease* the measured rate because recombination has meant that absorption in fewer particles is not compensated by the increased number of electron-hole pairs that are generated in each. Thus, on the basis of the optics alone more strongly absorbing particles are predicted to be less active than less strongly absorbing, larger particles.

#### 3.3.2. Effect of Milling on Photocatalytic Activity for Propan-2-ol Oxidation

**Figure 14:**
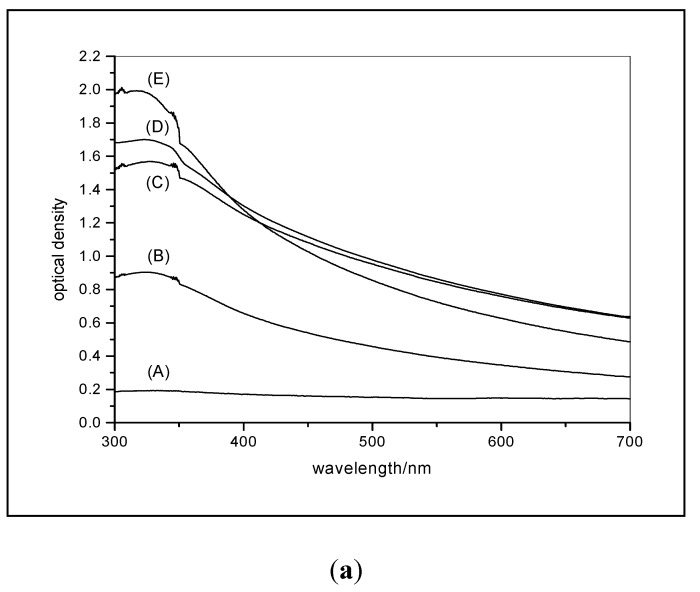

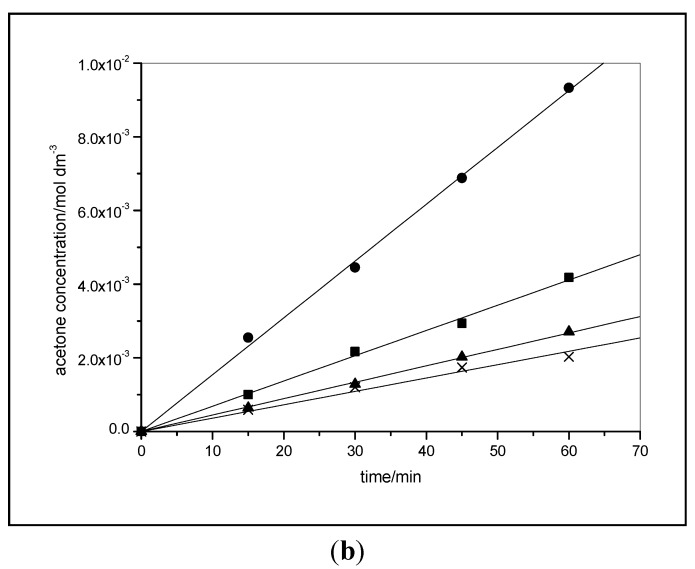
(**a**)Optical transmission curves measured on (A) unmilled 140 m^2^·g^−1^ rutile, and the same rutile milled for (B) 7.5 minute, (C) 15 minutes and (E) 30 minutes. Curve D was measured on a 15-minute milled and diluted sample that had been left to stand for the duration of a typical oxidation experiment prior to making the measurement; (**b**) The time dependence of propanone formation during the photocatalytic oxidation of propan-2-ol by the same 140 m^2^·g^−1^ rutile before sand-milling ●, and after milling for 7.5 ■, 15 ▲ and 30 ×, minutes. Reprinted from Egerton & Tooley, *J. Phys. Chem. B* **2004**, *108*, 5066–5072 [36] with permission.

The conclusion that improving catalyst dispersion, and decreasing the mean size of the agglomerated particles, reduces measured photocatalytic activity has been demonstrated for propan-2-ol oxidation using a high area rutile, 140 m^2^·g^−1^, with a line broadening size of 7–10 nm [36]. X-ray disc sedimentation measurements demonstrated that despite the XRD size of 7–10 nm the particle size of unmilled particles in suspension was greater than 40 nm and that vigorous milling was unable to reduce the size below 20 nm. The optical changes measured from 300 to 700 nm on diluted suspensions prepared by small-scale sand milling (probably less vigorous than used for the size-measurements) for increasing times are shown in Figure 14a. The significant turbidity between 400 and 700 nm is due to scattering and confirms that many particles must be of the order of 100 nm or more. The decrease in the turbidity at, e.g., 550 nm indicates that some of these large particles are broken down as milling times increase from 15 to 30 minutes. Correspondingly, the absorption below 400 nm increases as the milling time is extended. The effect of these changes in particle size are shown in Figure 14b. Similar results, a halving of the measured activity, have been measured for a rutile with a much larger primary particle size; ~200 nm by TEM and a surface area of 8 m^2^·g^−1^. A decrease in activity with milling has also been measured for P25 (Millenium), 10 m^2^·g^−1^ anatase (Huntsman), a low area rutile (Huntsman) surface treated with rutile, a 76 m^2^·g^−1^ anatase (Tayca) and high area rutile doped with iron [40].

#### 3.3.3. Effect of Milling on Photocatalytic Degradation of Salicylic Acid

**Figure 15:**
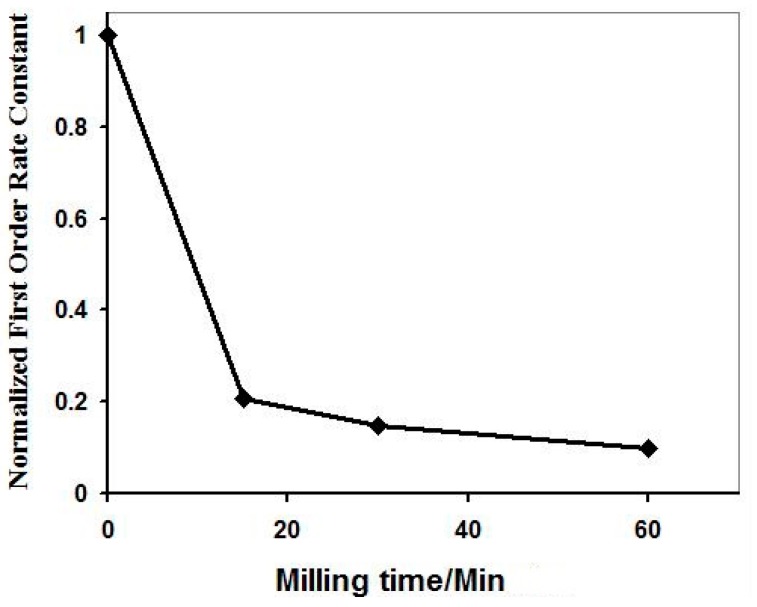
The effect increasing milling times on the first order rate constant for the degradation by high area rutile of 0.36 mM salicylic acid at pH 4. Reprinted from *J. Photochem. Photobiol. A* **2010**, *216*, 268–274 [40] with permission.

Propan-2-ol oxidation is believed to proceed by a hydroxyl radical mechanism and so the effect of milling on the photocatalytic degradation a reaction of salicylic acid has been investigated. (Preliminary experiments using neutral density filters confirmed the rate of this reaction varied as the square root of the UV intensity.) Once again, milling decreased the rate of reaction, as shown in Figure 15 [40].

#### 3.3.4. Effect of Milling on Photocatalytic Degradation of Dichloroacetic Acid

**Figure 16:**
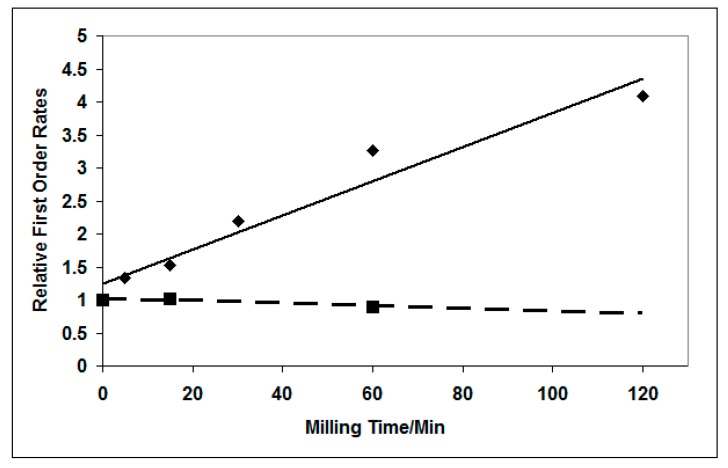
Effect of milling time on the DCA oxidation rate by a high area rutile ♦ and by P25 ■. Reprinted from *J. Photochem. Photobiol. A* **2010**, *216*, 268–274 [40] with permission.

The UV attenuation of suspensions of TiO_2_ in dichloroacetate increased with milling, as with propanol and salicylic acid. However, the rate of dichloroacetate degradation increased linearly with UV intensity, unlike the I^0^^.5^ dependency of rate found for propan-2-ol oxidation and salicylic acid degradation. Therefore the conclusion of Section 3.3.1, that more strongly UV-absorbing particles should be less active, is not necessarily valid for this system. The results shown in Figure 16 show that the photocatalytic activity for dichloroacetic acid degradation at pH 3 did *not* decrease as the catalyst suspension was milled. Therefore increased UV absorption by individual crystal should compensate for the reduction in the number of micro-reactors. For P25 rate was independent of milling time and for high area rutile of the rate actually increased [40].

## 4. Conclusions and Implications

UV absorption should be considered explicitly when considering TiO_2_ photocatalysis, because, in broad-band semiconductors, UV absorption initiates charge-carrier generation Despite many problems in their application, theories of light scattering and absorption have been of practical use in the design of inorganic UV–blocks for cosmetics and of opacifying pigments for paints. Both scattering and absorption depend on TiO_2_ particle size. However, the size that is relevant to photocatalysis in aqueous dispersions of TiO_2_ is *not* the size inferred from X-ray line-broadening or from surface area measurements. This is because these methods measure the size of primary particles, but particles in suspension are almost always aggregates or agglomerates of primary particles. It is the aggregated or agglomerated particles that control the optics.

The *protection* of commercial pigmented products such as polymers or paints, from UV is one demonstration of the practical significance of UV absorption by TiO_2_. Degradation is reduced because the reduction in the number of active photons is more important than the residual photocatalysis of surface-treated TiO_2_. Even for photocatalytic grades of TiO_2_, UV absorption reduces the number of active photons and can negate the benefits that have been claimed to result from the addition of photochemically active species to TiO_2_ suspensions. E.g., TiO_2_ can reduce the decolouration of dye residues by H_2_O_2_. Such effects should be explicitly considered in studies of hoped-for synergy.

Increased absorption by TiO_2_ particles may *decrease* measured photocatalytic rates. This is because charge-carrier recombination within particles may lead to the absorption of *n* photons by one photocatalyst particle being less effective than the absorption of 1 photon by each of *n* particles. Therefore, milling of photocatalysts to improve photocatalytic dispersion may reduce the measured rate of photocatalysis—as shown for the degradation of propanol, and salicylic acid. By contrast, milling does not decrease the measured rate of photocatalytic degradation of dichloroacetate; the difference is attributed to the fact that this reaction does not have an I^0.5^ dependence on the intensity, I, of the incident UV.

For reactions that do have an I^0.5^ intensity dependence, the effects of changes in dispersion may need to be considered. Sometimes, as when catalysts are immobilized on papers or fibres, these dispersion changes are deliberately induced. In many more cases they are overlooked, e.g., (i) if particles produced by a new synthetic route have a different degree of aggregation; or (ii) if deliberate changes in suspension pH alter surface potential and therefore flocculation; or (iii) if heat-treatment during doping increases aggregation. However, all such changes in particle dispersion may change the measured photocatalytic activity for reasons that are purely a consequence of the changed optics and suitable control experiments should be used.

## References

[1] Matsunaga T., Okochi M. (1995). TiO_2_-Mediated Photochemical Disinfection of *Escherichia coli* Using Optical Fibers. Environ. Sci. Technol..

[2] Maillard C., Guillard C., Pichat P. (1992). Comparative effects of the TiO_2_-UV, H_2_O_2_-UV H_2_O_2_-Fe^2+^ systems on The disappearance Rate of benzamide and 4-hydroxybenzamide in water. Chemosphere.

[3] Mills A., Belghazi A., Davies R.H., Morris S.A. (1994). Kinetic study of the bleaching of Rhodamine 6G photosensitized by titanium dioxide. J. Photochem. Photobiol. A.

[4] Fateh R., Dillert R., Bahnemann D. (2014). Self-cleaning properties, mechanical stability, and adhesion strength of transparent photocatalytic TiO_2_-ZnO coatings on polycarbonate. ACS Appl. Mater. Interfaces.

[5] Hughes W. (1970). Phodegradation of paint films containing TiO_2_ pigments. Xth FATIPEC Congress 1970.

[6] Allen N.S., McKellar J.F., Phillips G.O., Chapman C.B. (1974). The TiO_2_ photosensitized degradation of nylon 6, 6: Stabilizing action of manganese ions. J. Polym. Sci. C Polym. Lett..

[7] Turchi C.S., Ollis D.F. (1990). Photocatalytic degradation of organic-water contaminants—Mechanisms involving hydroxyl radical attack. J. Catal..

[8] Egerton T.A., King C.J. (1979). The Influence of Light Intensity on Photoactivity in TiO_2_ pigmented systems. J. Oil Colour Chem. Assoc..

[9] Bard A.J. (1978). Heterogeneous photocatalytic decomposition of saturated carboxylic acids on TiO_2_ powder decarboxylative route to alkanes. J. Am. Chem. Soc..

[10] Mills A., LeHunte S. (1997). An overview of semiconductor photocatalysis. J. Photochem. Photobiol. A.

[11] Glasstone S. (1962). Textbook of Physical Chemistry.

[12] Asahi R., Morikawa T., Ohwaki T., Aoki K., Taga Y. (2001). Visible-light photocatalysis in nitrogen-doped titanium oxides. Science.

[13] Vinodgopal K., Wynkoop D.E., Kamat P.V. (1996). Environmental photochemistry on semiconductor surfaces: Photosensitized degradation of a textile azo dye, acid orange 7, on TiO_2_ particles using visible light. Environ. Sci. Technol..

[14] Chen Y.S., Crittenden J.C., Hackney S., Sutter L., Hand D.W. (2005). Preparation of a novel TiO_2_-based p-n junction nanotube photocatalyst. Environ. Sci. Technol..

[15] Morawski A.W., Grzechulska J., Kalucki K. (1996). A new method for the preparation of potassium-pillared layered titanate applied in photocatalysis. J. Phys. Chem. Solids.

[16] Pelizzetti E., Carlin V., Minero C., Pramauro E., Vincenti M. (1992). Degradation pathways of atrazine under solar light and in the presence of TiO_2_ colloidal particles. Sci. Total Environ..

[17] Wang L., Egerton T.A. (2013). The influence of chromium on photocatalysis of propan-2-ol and octadecanoic acid oxidation by rutile TiO_2_. J. Photochem. Photobiol. A.

[18] Litter M.I. (1999). Heterogeneous photocatalysis-transition metal ions in photocatalytic systems. Appl. Catal. B Environ..

[19] Egerton T. (2011). Does photoelectrocatalysis by TiO_2_ work?. J. Chem. Technol. Biotechnol..

[20] Rayleigh L. (1871). On the light from the sky, its polarization and colour. Philos. Mag..

[21] Mie G. (1908). Articles on the optical characteristics of turbid tubes, especially colloidal metal solutions. Ann. Phys..

[22] Vos K., Krusemeyer H.J. (1977). Reflectance and electroreflectance of TiO_2_ single crystals. J. Phys. C Solid State Phys..

[23] Egerton T.A., Tooley I.R. (2012). UV absorption and scattering properties of inorganic-based sunscreens. Int. J. Cosmet. Sci..

[24] Robb J.L., Simpson L.A., Tunstall D.F. (1994). Titanium dioxide and UV radiation. Drug Cosmet. Ind..

[25] Ghosh A.K., Maruska H.P. (1977). Photoelectrolysis of water in sunlight on sensitized semiconductor electrodes. J. Electrochem. Soc..

[26] Mollers F., Tolle H.J., Memming R. (1974). On the origin of photocatalytic deposition of noble metals on TiO_2_. J. Electrochem. Soc..

[27] Martin C.A., Baltanas M.A., Cassano A.E. (1996). Photocatalytic reactors II. Quantum efficiencies allowing for scattering effects. An experimental approximation. J. Photochem. Photobiol. A.

[28] Romero R.L., Alfano O.M., Cassano A.E. (1997). Cylindrical Photocatalytic Reactors. Radiation absorption and scattering effects produced by suspended fine particles in an annular space. Ind. Eng. Chem. Res..

[29] Satuf M.A., Brandi R.J., Cassano A.E., Orlando M.A. (2005). Experimental method to evaluate the optical properties of aqueous titanium dioxide suspensions. Ind. Eng. Chem. Res..

[30] Marugan J., van Grieken R., Pablos C., Satuf M.L., Cassano A.E. (2013). Modelling of bench-scale photocatalytic reactor for water disinfection from laboratory-scale data. Chem. Eng. J..

[31] Toepfer B., Gora A., Li Puma G. (2006). Photocatalytic oxidation of multicomponent solutions of herbicides Reaction kinetics with explicit photon absorption effects. Appl. Catal. B Environ..

[32] Angel Mueses M., Machuca-Martinez F., Li Puma G. (2013). Effective quantum yield and reaction rate model for evaluation of photocatalytic degradation of water contaminants in heterogeneous pilot-scale solar photoreactors. Chem. Eng. J..

[33] Tunstall D.F., Hird M.J. (1974). Effect of particle crowding on scattering power of TiO_2_ pigments. J. Paint Technol..

[34] Martin C.A., Baltanas M.A., Cassano A.E. (1993). Photocatalytic reactors I. Optical behaviour of titanium oxide particulate suspensions. J. Photochem. Photobiol. A.

[35] Egerton T.A., Tooley I.R. (2014). Physical characterization of titanium dioxide nanoparticles. Int. J. Cosmet. Sci..

[36] Egerton T.A., Tooley I.R. (2004). Effect of changes in TiO_2_ dispersion on its measured photocatalytic activity. J. Phys. Chem. B.

[37] Ridley M.K., Hackley V.A., Machesky M.L. (2006). Characterization and Surface Reactivity of Nanocrystalline Anatase in Aqueous Solutions. Langmuir.

[38] French R.A., Jacobson A.R., Kim B., Isley S.L., Penn R.L., Baveye P.C. (2009). Influence of Ionic Strength, pH, and Cation Valence on Aggregation Kinetics of Titanium Dioxide Nanoparticles. Environ. Sci. Technol..

[39] Lee B.C., Kim K.T., Cho J.G., Lee J.W., Ryu T.K., Yoon J.H., Lee S.H., Duong C.N., Eom I.C., Ki P.J. (2012). Oxidative stress in juvenile common carp (Cyprinus carpio) exposed to TiO2 nanoparticles. Mol. Cell. Toxicol..

[40] Egerton T.A., Harrison R.W., Hill S.E., John A., Mattinson J.A., Purnama H. (2010). Effects of particle dispersion on the measurement of semi-conductor photocatalytic activity. J. Photochem. Photobiol. A.

[41] Fernando S.S., Christensen P.A., Egerton T.A., White J.R. (2007). Carbon dioxide evolution and carbonyl group development during photodegradation of polyethylene and polypropylene. Polym. Degrad. Stab..

[42] Egerton T.A. (1998). The modification of fine powders by inorganic coatings. Kona.

[43] Egerton T.A. (1997). Titanium Compounds, organic. Kirk-Othmer Encyclopedia of Chemical Technology.

[44] Egerton T.A., Tooley I.R. (2002). The surface characterization of coated titanium dioxide by FTIR spectroscopy of adsorbed nitrogen. J. Mater. Chem..

[45] Worsley D.A., Searle J.R. (2002). Photoactivity test for TiO_2_ pigment photocatalysed polymer degradation. Mater. Sci. Technol. Lond..

[46] Jin C. (1974). FTIR Studies of TiO_2_-pigmented Polymer Photodegradation. Ph.D. Thesis.

[47] Christensen P.A., Dilks A., Egerton T.A., Lawson E.J., Temperley J. (2002). Photocatalytic oxidation of alkyd paint films measured by FTIR analysis of UV generated carbon dioxide. J. Mater. Sci..

[48] Dillert R., Siemon U., Bahnemann D. (1998). Photocatalytic disinfection of municipal wastewater. Chem. Eng. Technol..

[49] Gupta V.K., Jain R., Agarwal S., Shrivastava M. (2011). Kinetics of photocatalytic degradation of tropaeoline 000 using UV/TiO2 in a UV reactor. Colloid Surf. A.

[50] Wu C.H. (2008). Effects of operational parameters on the decolourization of C.I. reactive red 198 in UV/TiO_2_ systems. Dyes Pigment..

[51] Sauer T., Neto C., Jose H.J., Moreira R.F.P.M. (2002). Kinetics of photocatalytic degradation of reactive dyes in a TiO2 slurry reactor. J. Photochem. Photobiol. A.

[52] Daneshvar N., Salari D., Khataee A.R. (2003). Photocatalytic Degradation of azo acid red 14 in water: Investigation of the effect of operational parameters. J. Photochem. Photobiol. A.

[53] Egerton T.A., Purnama H. (2014). Does hydrogen peroxide really accelerate TiO_2_ UV-C photocatalyzed decolouration of azo dyes such as Reactive Orange 16?. Dyes Pigment..

[54] Baxendale J.H., Wilson J.A. (1957). Photolysis of hydrogen peroxide at high light intensities. Trans. Faraday Soc..

[55] Kormann C., Bahnemann D.W., Hoffmann M.R. (1991). Photolysis of Chloroform and other organic molecules in aqueous TiO_2_ suspensions. Environ. Sci. Technol..

[56] Blake D.M., Webb J., Turchi C., Magrine K. (1991). Kinetic and Mechanistic Overview of TiO_2_. Sol. Energy Mater..

[57] Okamoto K., Yamamoto Y., Tanaka H., Itaya A. (1985). Kinetics of heterogeneous photocatalytic decomposition of phenol over anatase powder. Bull. Chem. Soc. Jpn..

